# Accurate Sizing and Resolution of Nominal 200 nm Diameter Polystyrene Nanospheres With Charge Detection Mass Spectrometry

**DOI:** 10.1002/smll.74148

**Published:** 2026-06-12

**Authors:** Veena S. Avadhani, Conner C. Harper, Evan R. Williams

**Affiliations:** ^1^ Department of Chemistry University of California Berkeley California United States

**Keywords:** CDMS, high‐throughput, nanoparticle, precision, sizing, TEM

## Abstract

Charge detection mass spectrometry (CDMS) with electrostatic ion traps has been used to characterize the mass distributions of a variety of nanoparticles that have masses ranging from ∼1 to 400 megadaltons (MDa). Here, samples of polystyrene nanospheres from three different manufacturers with diameters ∼200 nm and masses of several gigadaltons (GDa) were characterized by both CDMS and transmission electron microscopy (TEM). From the centroid masses of ∼2.320, ∼2.602, and ∼2.897 GDa, centroid diameters of 191.4, 199.1, and 204.8 nm were obtained, respectively. These diameters are within 1% of those obtained from TEM measurements. The size distributions of the individual components in a mixture of these three samples were readily resolved with CDMS but not with TEM. The greater measurement time required for a statistically meaningful measurement from TEM led to some particle shrinkage that was not uniform between particles. The width of the most monodisperse nanoparticle sample distribution indicates that CDMS could distinguish two nanoparticle samples that differed by just 2 nm in diameter at this particle size (1%). The high resolution, minimal sample preparation requirements, compatibility with automation, and now extended mass range make CDMS a powerful high‐throughput nanoparticle characterization tool for nanoparticles in biomedicine, advanced electronics, and material science.

## Graphical abstract

This study highlights the utility of charge detection mass spectrometry (CDMS) as a high‐throughput nanoparticle characterization tool. CDMS measurements of 200 nm polystyrene size standards are within 1% of those derived from transmission electron microscopy (TEM). Notably, a mixture of three ∼200 nm samples is readily resolved by CDMS but not by TEM, demonstrating excellent precision and accuracy of CDMS.

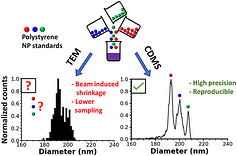

## 1 Introduction

The ability to change the size, shape and chemical composition of nanoparticles makes it possible to tailor physical properties that are optimum for different applications. Optical properties can be tailored by adjusting nanoparticle size or shape. For example, reducing the size of plasmonic nanoparticles results in blue shifts in the optical spectra [1]. For lipid nanoparticles, size can affect cellular uptake, leading to varied immune responses [2]. In nanofluid heat transfer applications, the size and shape of particles affect the thermal conductivity [3]. Nanoparticle composition also plays a crucial role in tailoring their suitability to specific applications. For example, metal nanoparticles are widely used in biosensing and medical imaging [4], carbon‐based nanoparticles are useful for energy storage [5], quantum dots are prominent in optoelectronics [6], and polymer [7] and lipid‐based nanoparticles are used in drug delivery systems [8, 9]. Polystyrene (or latex) is a common polymer nanomaterial with a surface that can be readily modified [10]. Latex beads have been used in biosensors [11], photonics [12], as size standards [13, 14], and models to study drug delivery [15] and ecotoxicological effects on cells [16]. Precisely controlled particle sizes are essential for applications such as semiconductor processing, aerosol property characterization, and electron microscope calibration [17, 18, 19]. Consequently, manufacturers produce polystyrene nanoparticles with precisely controlled sizes to serve as standards. These nanoparticles are typically characterized using National Institute of Standards and Technology (NIST) traceable methods and techniques [19].

Some of the common methods used to characterize nanoparticle size include photon scattering methods, such as dynamic light scattering (DLS) and small‐angle X‐ray scattering (SAXS) [20]. Microscopy‐based methods like scanning electron microscopy (SEM), transmission electron microscopy (TEM), and atomic force microscopy (AFM) provide information about size and shape at the individual particle level [21]. Mobility‐based methods, like differential mobility analyzers (DMA) or scanning mobility particle sizing (SMPS) instruments can be used to determine size and number concentration of particles in a sample [22]. Other methods include electro‐gravitational aerosol balance (EAB), scanning ion occlusion sensing (SIOS), nanoparticle tracking analysis (NTA), differential centrifugal sedimentation (DCS), resistive pulse sensing, and single particle inductively coupled plasma mass spectrometry (spICP‐MS) [23, 24, 25, 26]. These many techniques are based on different physical principles and can provide information about nanoparticle properties such as density, shape, and composition in addition to particle size. Several studies have compared the effectiveness of these different methods for size characterization [19, 27, 28]. Scattering‐based methods have the advantage that they are non‐destructive and analysis is done in solution, but these ensemble measurements tend to be biased toward larger particle sizes when the sample is polydisperse [29]. Use of particle dispersants in DLS can absorb light or hinder particle movement by modifying viscosity/ surface properties of the particles leading to inaccurate determination of hydrodynamic size [30]. In contrast, microscopy methods directly measure the size and morphology of individual particles. However, sample preparation, such as sample fixation on grids or introduction into high vacuum, can introduce artifacts. Determining the boundaries of the particle surfaces and challenges in automated analysis can also limit the number of particles analyzed, making these methods impractical for high‐throughput measurements. Multiple characterization methods, including SMPS, DLS, SIOS, NTA, and DCS, were systematically evaluated by Bell et al. [28] for their relative accuracy and precision for sizing 100–400 nm silica nanoparticles, with TEM serving as the reference standard. Information about sample preparation, time and cost of using these techniques were also compared. SIOS was the least expensive method but was least precise. SMPS results were the most reproducible but required a large sample volume (200 mL) which may preclude its use in many applications. All techniques, except TEM and NTA, had larger arithmetic means than modes. While the mode can serve as a measure of the most probable particle size, the difference between the arithmetic mean and mode provides a measure of the asymmetry of the distribution. For techniques like DLS, SIOS, DCS and SMPS, this discrepancy arises due to bias toward measuring larger particles/aggregates [31, 32]. For a particle with a mean diameter of 376 nm measured by TEM, diameters obtained from the other techniques ranged between 344 and 400 nm [28]. These values are all within one standard deviation (29 nm) of the distribution measured by TEM. However, the spread of the size distributions differed substantially between the methods. For example, the full‐width half maximum (fwhm) of a size distribution for the same sample measured by DLS and by TEM was 189 and 31 nm, respectively.

While TEM is generally considered to be the gold standard method for measuring particle sizes, the measurements are relatively time‐consuming and expensive. Mulholland et al. compared traceable measurement techniques at different national metrology institutes that provide calibration standards for spherical nanoparticles [19]. They used DMA, AFM, SEM, and EAB to characterize 100 nm polystyrene latex particles. The number‐average diameters obtained from all four techniques agreed to within 3%, with SEM giving the smallest mean value. The diameter distributions, however, differed substantially in width. Electron microscopy gave the broadest distribution (3.7 nm), compared with 0.39 nm for EAB and 1.2 nm for DMA. Because the EAB mean and its associated uncertainty show very limited overlap with the corresponding ranges from the other techniques (as little as 0.1 nm overlap with DMA), this discrepancy could become significant if different metrology institutes rely on different methods for certifying standard particle sizes. Potential reasons for the differing averages and uncertainties include bead shrinkage under electron beam for SEM, loss of sphericity of particles due to adhesion‐force distortion for AFM, uncertainty in slip correction factor for DMA, and inadequate estimate of surface residues postevaporation of aerosolized particles for EAB and DMA.

Charge detection mass spectrometry (CDMS) is a relatively new method that can characterize individual nanoparticles by directly measuring their mass and charge [33, 34, 35, 36, 37, 38, 39]. Mass is a fundamental property of a particle that can be related to physical size if information about the shape and density of the nanoparticle is known. Charged particles are formed directly from solution with electrospray ionization (ESI), and the extent of nanoparticle charging can provide information about the nanoparticle shape, surface properties, and density [40, 41, 42]. Advantages of CDMS to characterize nanoparticle distributions include high precision/accuracy, and many thousands of particles can be rapidly measured with the ability to automate the mass and particle size analysis. Small uncertainties in measured particle mass translates to even lower relative uncertainties in particle size. For example, even a relatively large mass error of 1.5% corresponds to only a 0.5% uncertainty in diameter for a spherical particle [43]. Using dynamic energy measurement methods with CDMS, the mass measurement precision improves with increasing mass (and measurement time) making this a promising method for characterizing a wide range of particle sizes [44, 45]. CDMS has been applied to many different types of particles including polymers [45, 46, 47], viruses [42, 48, 49, 50], virus‐like particles [51, 52, 53, 54, 55, 56, 57, 58], protein aggregates [59, 60, 61, 62, 63, 64], lipid nanoparticles [65, 66], water nanodrops [67, 68, 69, 70, 71], and synthetic particles [40, 41, 43, 72, 73, 74, 75]. Prior work using cone‐trap CDMS showed that accurate mass measurements could be made for nanoparticles with masses below ∼800 MDa corresponding to dimers of 100 nm polystyrene beads [43]. Here, the ability to measure accurate and precise size distributions from mass measurements of nominal 200 nm polystyrene nanospheres obtained from three different manufacturers that have masses in the GDa range is demonstrated along with separation of these samples in a mixture despite their similar sizes.

## 2 Experimental Section

### 2.1 Materials

Three different polystyrene nanoparticles with sizes around 200 nm were used in these experiments. A 200 nm polystyrene size standard nanosphere (1% w/v aqueous suspension – 2.27 × 10^12^ particles/mL) was obtained from Thermo Scientific (catalog number: 3200 and 3200A). The manufacturer certified mean diameter is 202 ± 4 nm (certified batch number: 3200‐010) determined using TEM from NIST reference materials. Another polystyrene bead sample was acquired from Polysciences (catalog number: 07304‐1) as 2.5% solids (w/v) aqueous suspension (5.68 × 10^12^ particles/mL). The manufacturer specified mean dimension is 180 nm with an 8% coefficient of variance. A third sample of ∼200 nm nanospheres from Colloidal Metrics was obtained from Professor Juan Fernandez de la Mora (Yale University). These beads were synthesized using ∼0.99% latex solids (86% polystyrene and 14% polymethyl methacrylate by mass) and ∼0.008% non‐polymer solids in an aqueous suspension [76]. The concentration of this sample was not known. Based on the relative particle detection rates of the three samples, for which the concentrations of two are known, the concentration of the Colloidal Metrics sample was estimated to be ∼3 × 10^12^ particles/mL (SI). Samples for CDMS analysis were prepared by diluting each of these samples in a 1:200 ratio with 0.5% aqueous acetic acid. A mixture of nanospheres from Polysciences (P) and Colloidal Metrics (CM) was made by mixing equal volumes of their 1:200 diluted solutions. A mixture of P, CM, and nanospheres from Thermo Scientific (T) was made using 1:1:2.5 ratio.

### 2.2 Charge Detection Mass Spectrometry

A custom‐built electrostatic ion trap charge detection mass spectrometer that is described in detail elsewhere was used for these measurements [43]. Gas‐phase charged polystyrene nanoparticles were generated using nanoelectrospray ionization (nESI) from borosilicate emitters by applying +1.7 to +2 kV to a platinum wire that was in contact with the sample solution. Emitters with tip diameters of either ∼5 or ∼10 µm were used. The emitters were positioned ∼3 mm from the sample inlet of the CDMS instrument. Ions passed through a heated metal capillary inlet, ion funnel and were then trapped in a quadrupole mass filter before being accelerated and introduced into an electrostatic conetrap where they were trapped for 100 ms. Detailed information about specific instrument parameters is provided in Table S1. The trapped ions oscillate inside the trap and induce a current pulse every time they pass through a conductive cylinder in the center of the trap. This induced current is proportional to the charge on the ions. Short‐time Fourier‐transform (STFT) analysis of time domain data was performed using a 50 ms window length, 5 ms stepsize, and 500 ms zerofills. This method enables dynamic tracking of mass, charge, and energy of each ion throughout the entire measurement period [44, 45, 77]. Mass measurements were performed with just a single ion in the trap owing to the low concentrations (∼10^9^–10^10^ particles/mL) that were used to avoid clogging of the nESI emitters.

### 2.3 Transmission Electron Microscopy

Polystyrene nanoparticle samples were diluted in milliQ water by a factor of 50 (∼10^11^ particles/mL) for TEM imaging. Formvar/carbon‐coated 300 mesh copper grids from Ted Pella Inc. (Redding, CA, USA) were used as substrates for nanoparticle samples. Each grid was made hydrophilic with an easiGlow (Pelco) benchtop glow discharge unit. A 5 µL sample volume was placed on each grid for 2 min post hydrophilization. Filter paper was used to remove excess fluid on the grid. These dried grids were then imaged using a Tecnai 12 120 kV transmission electron microscope. Images were recorded using a Rio 16 CMOS with DigitalMicrograph software (Gatan Inc.). Images used for particle diameter measurements were captured at a magnification that resulted in a resolution of 1.41 pixel/nm. Samples were irradiated for 12 min and images were captured at 2, 5, 7, 9, and 12 min. The starting point of 2 min corresponds to the time needed to find an ideal frame at desired magnification and focus the beam to achieve good resolution. The electron beam spot size was increased at constant current in some cases to test the impact of reduced intensity beam conditions on the extent of nanosphere shrinkage. The resulting images were analyzed using FIJI/ImageJ version 1.53v. An automated analysis method based on Hough Circle Transform was used to identify spheres and fit circles on them to determine radius of the nanoparticles [40]. Images showing circle fits (Figure S1) and details on parameters used for implementing the transform are provided in the Supplementary Information.

## 3 Results and Discussion

### 3.1 Characterizing Nanoparticles by CDMS and TEM

**FIGURE 1:**
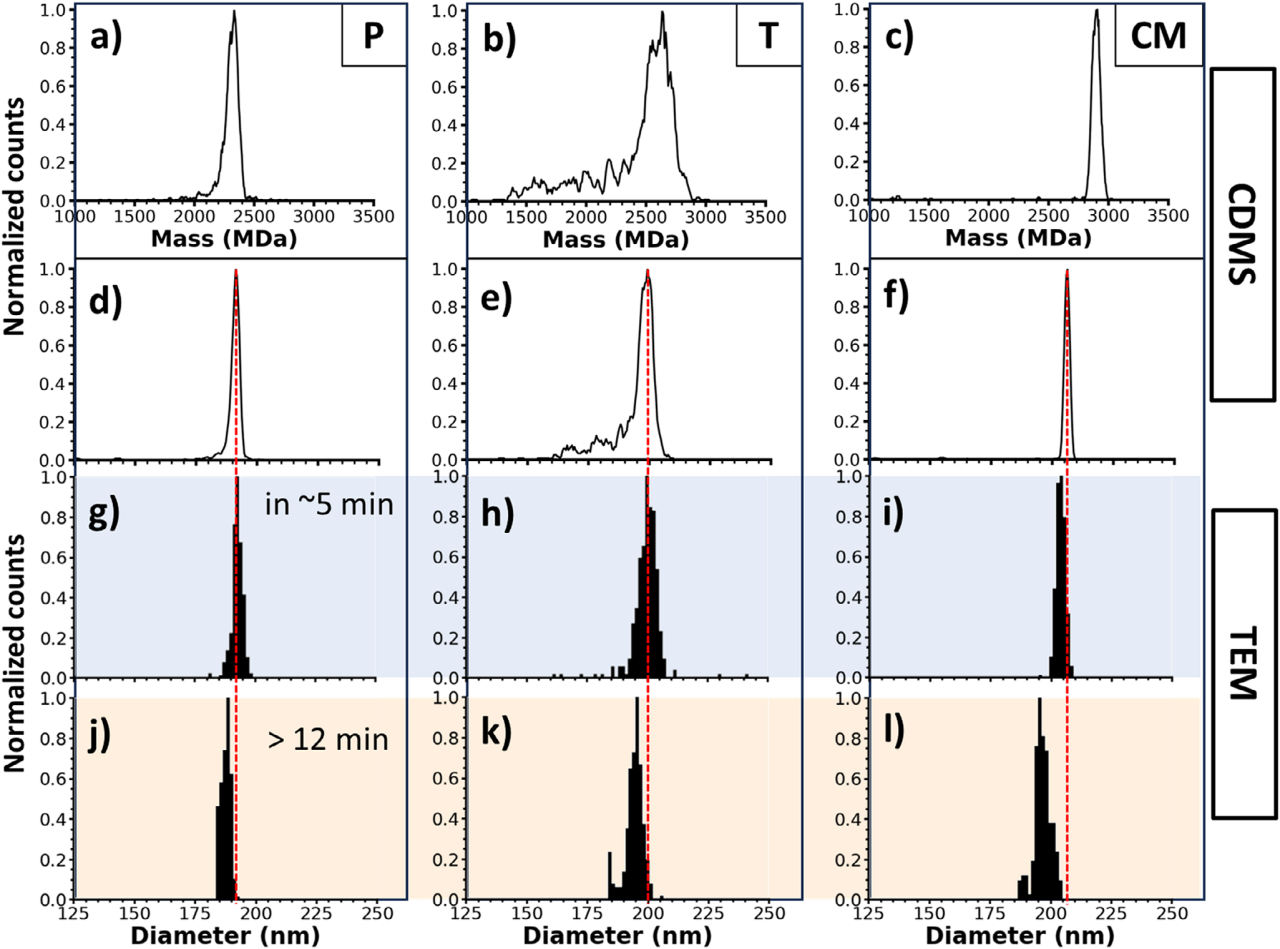
Mass histograms from CDMS measurements of (a) P, (b) T, and (c) CM polystyrene nanoparticles; (d–f) resulting diameter distributions (bin size = 0.5 nm) using mass data for P, T and CM nanospheres, respectively; (g–i) diameter distributions (bin size = 1.4 nm) from analysis of TEM images captured in ∼5 min; and (j–l) more than 12 min for all the three types of nanoparticles. The dashed red line through the diameter distributions is centered on the maximum in the CDMS diameter data for comparison to the TEM data.

Three commercial samples of polystyrene beads with nominal diameters ∼200 nm were characterized using both CDMS and TEM. CDMS mass histograms of these samples are shown in Figure 1a–c and correspond to measurements of 1146, 680, and 566 charged particles for samples P, T, and CM, respectively. The mass histogram of the CM sample is narrow and symmetrical whereas both samples P and T have peaks that tail off to lower mass. The low mass tailing in sample T is similar to that of a 100 nm polystyrene nanosphere standard [40] from the same manufacturer and appears to be characteristic of these samples. A Savitzsky–Golay filter was used to smooth these data, and a Gaussian fit was used to determine the mass of the most abundant particles in these distributions (centroid of the fit). These masses are ∼2.320, ∼2.602, and ∼2.897 GDa for samples P, T, and CM, respectively.

To meaningfully compare these data with TEM results, the masses of the individual particles are converted to particle diameters via the known shape and density of the particles. TEM data confirm that these particles are spheres. P and T nanoparticles have a manufacturer‐reported density of 1.05 g/mL. However, CM nanoparticles are composed of 86% polystyrene (1.0502 g/mL density) and 14% polymethyl methacrylate (1.185 g/mL density) by mass. The density of these particles was estimated from the weighted average of the densities of constituent bulk components (1.069 g/mL) as was done previously [40]. From the density of these samples, a particle diameter distribution can be obtained from the measured mass data (Figure 1d–f). The maxima of Gaussian fits for these distributions are 191.4 nm (2.9 nm fwhm), 199.1 nm (7.0 nm fwhm), and 204.8 nm (1.8 nm fwhm) for samples P, T, and CM, respectively. CM is the most monodisperse sample with a coefficient of variation (CV), defined as the ratio of the standard deviation to the mean diameter, of less than 0.5%. Fernandez de la Mora [76] characterized sample CM using a high‐resolution differential mobility instrument and obtained a mean diameter of 209.2 nm with 1.09 ± 0.05% fwhm. This diameter derived from ion mobility data is slightly higher than the mean diameter of 204.7 nm obtained from CDMS. This small difference can be attributed to not having a calibrant at this size leading to ∼2% uncertainty in the DMA mobility scale [76, 78]. However, the similarity between the widths of the size distributions, 1.09% fwhm for DMA and 0.88% fwhm for CDMS, indicate that this sample has unusually low polydispersity. To determine the reproducibility of the diameter distributions obtained from CDMS mass distributions, three separate measurements of sample P were performed using different nESI emitters for each measurement. The standard deviation of the resulting centroids was 0.12 nm (0.06%), which is a lower value than was measured for all of the techniques reported by Bell et al. [28], including TEM, which had a standard deviation of 2.9 nm (0.9%).

Results from TEM measurements of these same three samples are shown in Figure 1g–i. The histograms consist of measurements of ∼300 nanoparticles each. The centroids of Gaussian fits for diameter distributions (bin size = 1.4 nm) obtained from the TEM measurements are 192.8 ± 4.6 nm (fwhm), 200.5 ± 8.1 nm, and 203.8 ± 4.7 nm for samples P, T, and CM, respectively. These values are in excellent agreement with the diameters obtained from CDMS mass measurements, with a difference in diameter of less than 1.0% for each of the three samples. Some variance is expected due to the limited number of particles sampled. Importantly, there does not appear to be a systematic difference between the two techniques. The manufacturer certified NIST traceable mean diameter of sample T is 202 ± 5.1 nm (standard deviation). This value is slightly higher than value obtained from Gaussian distributions of the CDMS values (199.2 ± 7.0 nm) and from our TEM (200.5 ± 8.1) measurements, but these measurements are well within the uncertainty of the certified value. Filipe et al. [79] reported mean values of 218 and 200 nm for T sample using DLS and NTA, respectively. The higher mean value from DLS likely reflects inherent bias toward larger particles [29]. While NTA provides an accurate mean diameter, the associated peak width (fwhm ≈ 70 nm) is too broad to resolve the discrete size differences between P, T, and CM nanoparticles. A comparative analysis of size measurements obtained via different analytical techniques for 100 and 200 nm T samples is provided in Table S2. The mean diameters measured by CDMS and TEM for this sample are 193.8 and 200.2 nm, respectively. The lower mean from CDMS reflects a pronounced tail toward smaller diameters (Figure 1e), likely arising from malformed or incompletely formed particles in the sample that are not captured by the automated TEM image analysis. Consequently, mean values may not be the most appropriate metric for comparing the two techniques. A more comprehensive comparisons of mean, mode, and Gaussian fit centroids for all PS beads are provided in Table S3.

Prior CDMS measurements of 100 nm polystyrene particle standards showed excellent agreement with results from TEM measurements from the 100 nm T sample, but TEM measurements of the 100 nm CM sample indicated that the diameters were ∼5.5% lower than the value obtained with CDMS [40]. This difference was hypothesized to be due to shrinkage of the mixed polymer nanospheres from CM due to effects of the energetic electron beam in the TEM measurements. This hypothesis was supported by clear observations of shrinkage of particles in the current study. The extent of shrinkage was obtained from TEM measurements that were done over a longer time frame (>12 min). The centroids of Gaussian diameter distributions from these measurements for the P, T, and CM samples were 187.1 nm ± 5.5, 195.5 nm ± 6.4, and 197.1 nm ± 6.8 nm, respectively (Figure 1j–l). These diameters are ∼2.5%–3% lower than the diameters obtained from shorter TEM measurements and from CDMS mass measurements.

### 3.2 Electron Beam‐Induced Shrinkage of Polystyrene Beads

Watson and Grube [80] first cautioned about the use of latex beads as calibration standards in electron microscopy in 1952, and several studies on electron beam interactions with latex have been done [81, 82, 83, 84, 85]. Damage by the beam can be initiated by atom displacement due to momentum transfer from the electron beam or collective atom migration caused by secondary effects, such as induced electric field [82]. The irradiation can also introduce defects in the material allowing diffusion of material to form a neck at the point of contact between beads [83]. This effect, known as sintering, was observed in most of our TEM images that were captured after >10 min of irradiation. Figure S2 shows 2 example images of CM beads with stringy attachments between the beads caused due to sintering. This observation led us to further explore effects of the electron beam on particle size.

**FIGURE 2:**
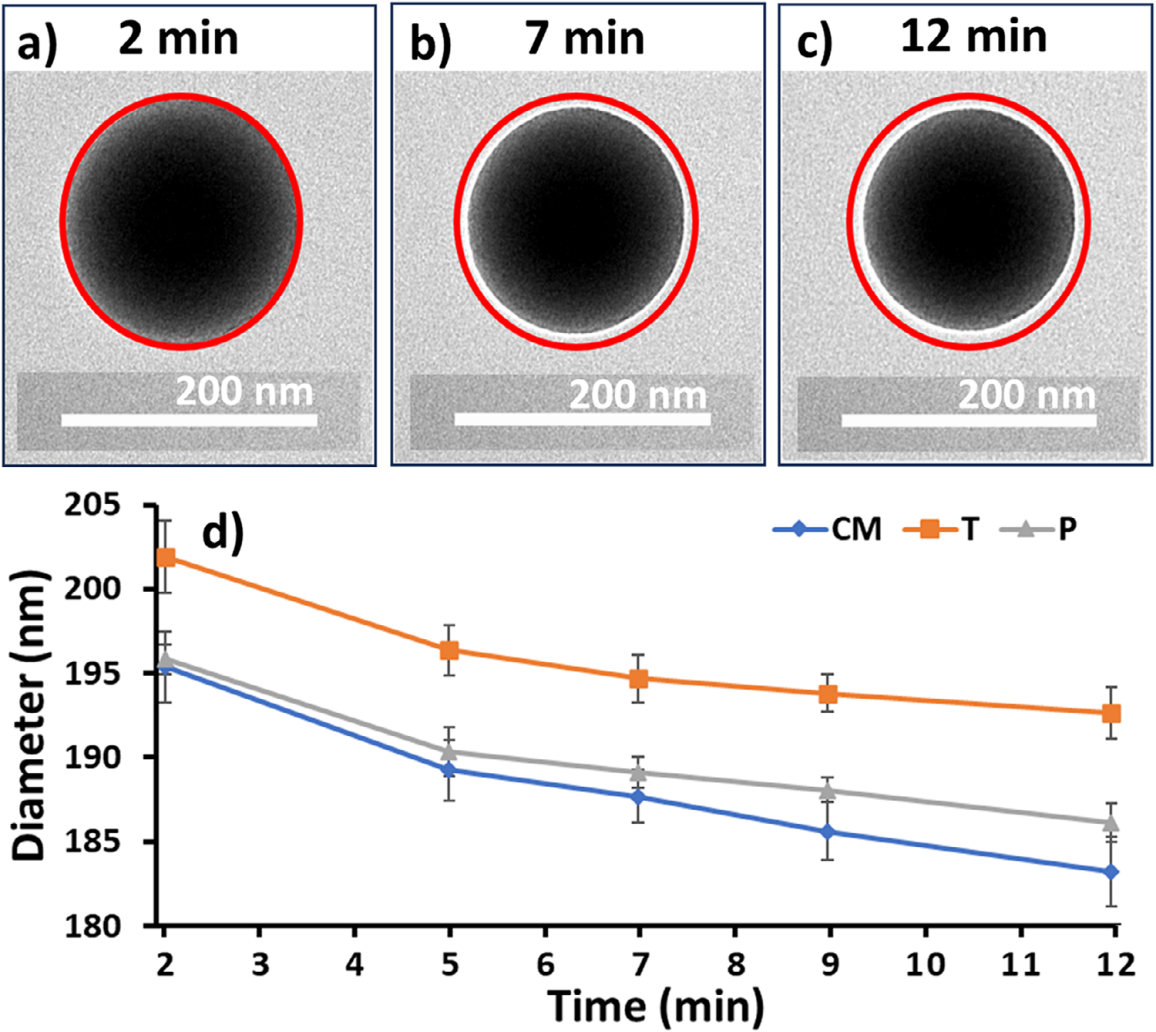
TEM images of the same P polystyrene bead in focus after electron beam irradiation for (a) 2, (b) 7, and (c) 12 min. The overlaid red circles show the size of the nanosphere at 2 min and show the effects of shrinkage with irradiation time corresponding to (a) 184.3, (b) 170.5, and (c) 166.5 nm; (d) change in diameter as a function of beam irradiation time for all the three nanoparticle samples. The error bars represent the standard deviation of multiple manual measurements of a single bead in focus. Note: The P nanoparticle in (d) is not the one displayed in (a–c).

A single polystyrene nanoparticle of sample P was irradiated for 12 min by a 120 kV electron beam using a typical current of 1 µA. Images were obtained between 2 and 12 min. The initial measured diameter of this particle was 184.3 nm (Figure 2a) but the measured diameter decreased to 170.5 and 166.5 nm at 7 min (Figure 2b) and 12 min (Figure 2c), respectively. Similar shrinkage as a function of irradiation time was observed for particles from both of the other two samples, and these data are shown in Figure 2d. After 12 min, these nanoparticles reduced in size by 4.9%, 4.6%, and 2.6% for P, T, and CM, respectively. The decrease in size is more significant in the first few minutes of the beam interacting with the nanoparticle. Milder beam condition with larger spot size (lower beam current per unit area) also resulted in a shrinkage of a different P bead from 187.9 to 166.3 nm (Figure S3). This observation of bead shrinkage independent of the radiation dose over the limited range used is consistent with the trends reported by Jung et al. [85]. Thus, we conclude that a prior inconsistency between CDMS diameter measurements for the 100 nm CM beads and TEM measurements was almost certainly related to particle shrinkage in the electron beam as was originally hypothesized. Care should be taken to minimize the time a sample is exposed to an electron beam in order to obtain accurate measurements.

### 3.3 Characterization of Polystyrene Bead Mixtures

**FIGURE 3:**
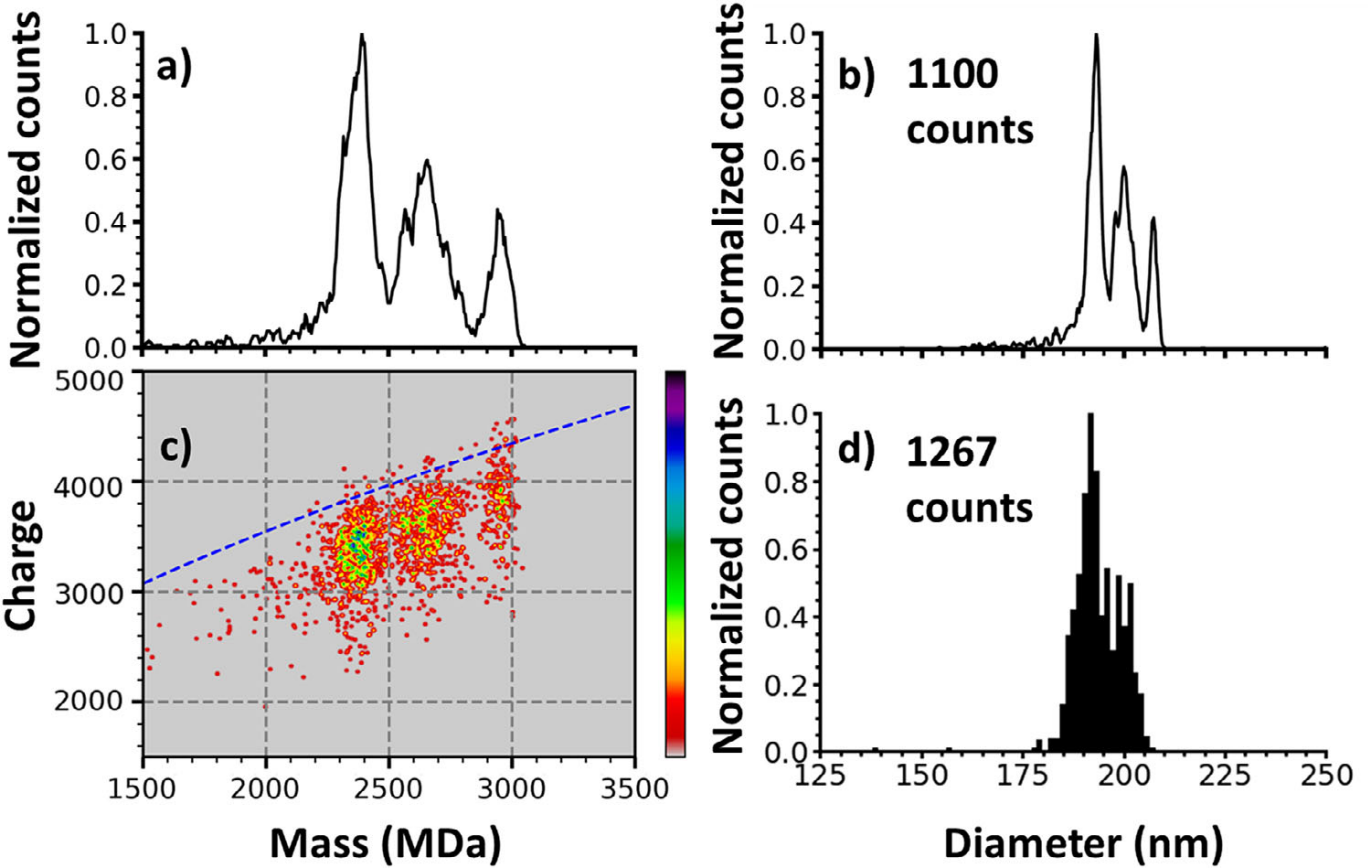
(a) Mass histogram from CDMS measurement of a 1:2.5:1 volume mix of P, T, and CM polystyrene nanoparticles; (b) corresponding diameter distribution (bin size of 0.2 nm) using the mass data; (c) 2D mass vs. charge histograms portraying clear distinction between different types of particles. The blue line represents Rayleigh charge limit for a water droplet of given mass; and (d) diameter distribution of the same mixture obtained from TEM measurements using a bin size of 1.4 nm. Comparison of (b) and (d) highlights the ability of CDMS to distinguish between the closely spaced nanoparticles that can be more challenging to distinguish by TEM. To reduce sampling gaps in the TEM data that had a 0.7 nm/pixel resolution, a bin size of 1.4 nm was used. Because CDMS resolution is not limited by digital noise, a small bin size was used.

To evaluate the ability of CDMS to separate similar‐sized nanoparticles, a mixture of the three samples, P, T, and CM was prepared in a 1:2.5:1 ratio. This mixture was analyzed using CDMS and a mass histogram of this mixture is shown in Figure 3a. These data were transformed into particle diameters (Figure 3b) using a density of 1.0502 g/mL and show three centroids at 192.8 ± 3.6 nm, 200.0 ± 4.9 nm, and 207.2 ± 2.2 nm. These data demonstrate that these three different nanoparticle samples can be clearly distinguished with CDMS. A single density value of 1.0502 g/mL was used to convert particle mass to diameter. If a density appropriate for the CM nanoparticles of 1.069 g/mL were used [40], the CM nanoparticles’ distribution centroid would be at 206 nm, corresponding to only a 0.6% difference in diameter. However, the diameter values from this measurement on the mixture are ∼0.8% higher than those from the measurement of the individual particles (Figure 1d–f).

The small discrepancy in the diameters measured individually and in a mixture is likely due to some excess water, ions, and/or surfactant adhering to these particles when formed from the mixture owing to the different emitter tip sizes that were used. The 2D charge vs. mass plot (Figure 3c) shows that these particles formed from the mixture are charged to ∼89% of the Rayleigh limit calculated for a water nanodrop as a function of mass (Figure 3c, blue line). In contrast, the polystyrene beads in the measurements of the individual samples were charged to ∼77% of the Rayleigh limit (Figure S4). The ∼12% higher charging of the individual components in the mixture and the slightly higher measured masses both indicate that a small amount of solvent, ions, and possibly additional surfactant remained with the nanoparticles from the ESI process in the measurements of the mixture [40]. ESI emitters with 5 µm diameter tips were used for the mass measurements of the individual components whereas a 10 µm ESI emitter was used for the measurements of the mixture. An emitter with a larger tip diameter was used for the mixture to overcome clogging of the emitter tip that occurred when using an emitter with a 5 µm diameter tip. Emitters with larger tip diameters produce larger droplets that can lead to less efficient desolvation and more adduction to particles. However, they also reduce effects of clogging. The three different nanoparticle samples were likely stabilized using different surfactants or polymers. Mixing these samples may have led to increased interactions that triggered precipitation in the confined space of the emitter tip. Emitters with larger tip diameters have higher flow rates and lower sample confinement that may reduce the time for particle aggregation to occur. Additional instrumental parameters could be optimized to reduce the extent of solvation, but the slightly higher average mass, diameter, and charge obtained for the mixture of polystyrene beads is still within the full width half maximum of the distributions obtained from individual experiments.

The three different samples can be clearly distinguished in the CDMS spectrum of the mixture despite the spread in average particle diameters of only ∼7%. Filipe et al. [79] demonstrated that even a mixture of 100 and 200 nm PS beads was not resolved using DLS. While NTA is capable of distinguishing 100 and 200 nm populations from a mixture, the peak width (fwhm ≈ 70 nm) observed for 200 nm particles confirms that NTA lacks the precision required to resolve the closely spaced P, T, and CM bead sizes. Even with TEM, the three samples were not differentiated from one another (Figure 3d). CDMS mass values are a continuum, making it possible to optimize bin sizes in the diameter histograms depending on the number of charged particles measured. A bin size of 0.2 nm was used for CDMS data shown in Figure 3b. In contrast, the TEM images have a resolution of 0.7 nm/pixel, and the automated measurement software output results in integer value of pixels. Thus, the bin size for TEM‐derived diameters with this method is limited to multiples of 0.7 nm. To ensure statistical continuity and avoid appearance of empty bins, a 1.4 nm bin size was used for the TEM data shown in Figure 3d. However, even with a TEM image resolution of 0.7 nm, these three samples are not resolved indicating that bin size does not contribute to the inability of TEM to resolve these samples. The TEM results are from 1267 individual particles, which is comparable to the 1100 particles measured with CDMS. All the images were captured within a span of 7 min of irradiation to reduce the impact of shrinkage on the measurements. However, the inability to distinguish between different samples may be due to varying extents of shrinkage that can occur for individual particles exposed to the electron beam. An illustration of this effect is shown in Figure S5 where two images of this mixture obtained at 2 and 12 min contain multiple beads that underwent shrinkage after exposure for 10 additional minutes. The diameter of one of the nanospheres decreased by 5.2% whereas another decreased by 3.5%. Optimizing TEM conditions used to analyze this mixture may lead to more separation of these samples but results from CDMS show that this method can readily separate the individual constituents in a mixture of similar sized nanospheres with little optimization.

### 3.4 Particle Resolution Achievable With CDMS

Dynamic tracking of analyte mass in CDMS makes it possible to obtain a measure of mass uncertainty from the many repetitive measurements that are made throughout the entire time a particle is trapped. The standard error in mass measured in shorter time segments spanning the entire trapping time leads to a mass precision of ∼0.6% for a single 200 nm PS bead that is trapped for 100 ms. This mass uncertainty translates to ∼0.2% or 0.4 nm uncertainty in the value of diameter that is obtained for each particle from these measurements. In principle, the uncertainty can be further improved by using longer measurement times. For example, a 5 s measurement would lead to ∼0.08% mass uncertainty, and hence, ∼0.03% diameter uncertainty or a fraction of an angstrom [77]. However, this level of mass precision is not useful especially when other factors, such as solvent or surfactant adduction play a much larger, albeit still small, effect on the diameter of a particle determined from its mass. Thus, the widths of the diameter distributions for the three samples determined by CDMS is limited not by instrument resolution, but by the intrinsic size distribution of the samples and any associated adduction.

**FIGURE 4:**
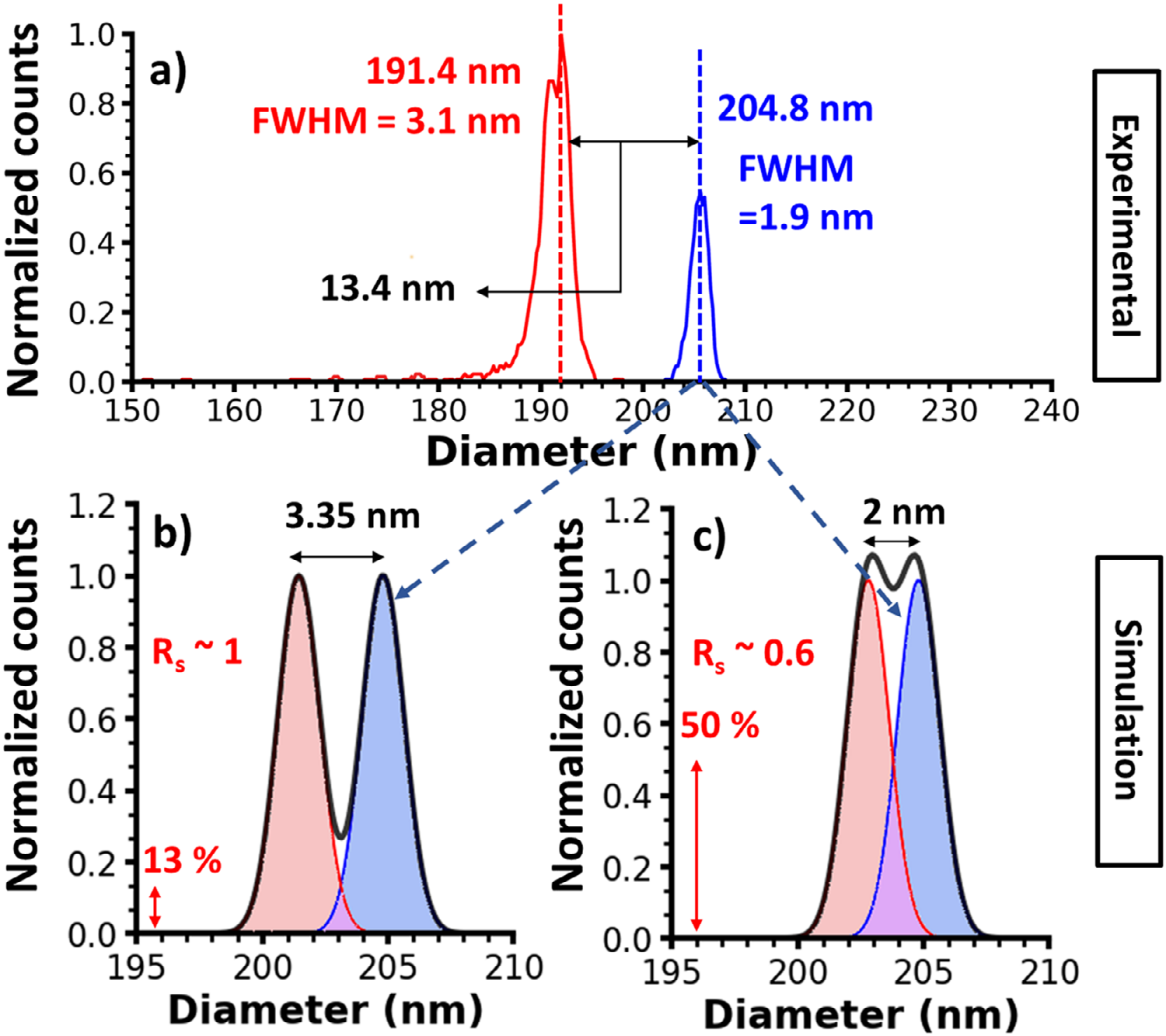
(a) Diameter histogram of a 1:1 volume mix of P (red) and CM (blue) nanoparticles obtained using CDMS. Both the particle samples are baseline resolved using CDMS in this mixture; (b) simulated results for two samples with diameter distributions as monodisperse as the CM nanoparticles where CDMS can (b) easily resolve ∼200 nm diameter particles that are 3.35 (1.7%) nm apart with reliable quantitation of the two; and (c) resolve ∼200 nm diameter particles that differ by only 2 nm (1%) using peak fitting and sufficient ion counts. The Gaussian peak resolutions are 1 and 0.6 for (b) and (c), respectively.

The size distribution of sample T is the broadest of the three samples. CDMS results for a 1:1 mix of P and CM led to two diameter distributions that are baseline resolved (Figure 4a). The centroids of these diameter distributions (using a density of 1.0502 g/mL) are 191.4 and 204.8 nm, values that are the same as those measured for the individual particles (Figure 1d,f, respectively). The same emitter tip diameter (5 µm) was used for this mixture as it was for the individual sample measurements, and this emitter size is sufficiently small to allow efficient desolvation of these analytes. The charge states are also similar to those of the individual measurements (Figure S6) and these values are lower than the mixture of the three‐component mixture where a larger (10 µm) diameter emitter was used, providing additional evidence for differing extents of desolvation associated with emitter size.

The CM sample has the narrowest diameter distribution (1.9 nm fwhm) leading to a diameter resolution, d/∆d, of 204.8/1.9 = 108. This number reflects sample‐limited resolution and is not a measure of the intrinsic performance of the instrument. To evaluate the ability of CDMS to differentiate between closely sized nanoparticles that have similarly low polydispersity, two of the same Gaussian functions used to fit the CM size distribution were separated by 3.35 nm and centered at ∼203 nm. This ∼1.7% difference in diameters leads to peaks that overlap by ∼13% above baseline (Figure 4b). Using a standard chromatographic definition of resolution (R_s_) [86, 87], the two peaks have R_s_ ≈ 1. At this resolution, the valley between the peaks (solid black lines in Figure 4b) is below 50% of the peak height, making possible accurate determination of the fwhm for both peaks. Consequently, an R_s_ of approximately 1 is generally considered sufficient for reliable quantitation. With this definition of resolution, CDMS can distinguish two similarly monodisperse nanoparticle samples that differ in diameter by only ∼1.7%. If the two distributions are separated by only 2 nm (∼1%), the peaks overlap by ∼50% above baseline (Figure 4c). In this case, the peaks could still be distinguished provided that a sufficient number of particles are measured, but R_s_ must be estimated by peak fitting and simulation (R_s_ ≈ 0.6 in this example). Much improved separation would, of course, be achievable for samples with even lower polydispersity.

TEM data reported by Bell et al. [28] have a standard deviation of 2.9 nm (0.9%) for nominal 100–400 nm particles. This level of variability suggests that distinguishing two samples separated by 2 nm (0.97%), as in Figure 4c, would be difficult by TEM if similar measurement precision applies at 200 nm. Moreover, the samples must be stable under the electron beam. For analytes that undergo beam‐induced shrinkage, as was the case in our study, the effective measurement precision is further reduced.

Mulholland et al. [19] reported coefficients of variation (CVs) of 1.6%, 1.7%, and 2% for a 100 nm polystyrene standard measured by SEM, AFM, and DMA, respectively. In contrast, CDMS resulted in a CV of 0.39% for the CM sample, underscoring the highly monodisperse nature of this nanosphere sample, which may prove useful for some applications and for benchmarking the performance of other methods. Overall, the ability of CDMS to resolve two monodisperse nanoparticle populations differing by only 2 nm at 200 nm size demonstrates a level of resolution and reproducibility that exceeds that of current state‐of‐the‐art particle sizing techniques.

## 4 Conclusions

A wide range of techniques are available for nanoparticle size characterization, each offering different levels of precision, accuracy, and cost. CDMS provides excellent precision and accuracy that is comparable to or exceeds that of TEM, the current gold standard of particle size measurements. Because converting CDMS mass distributions to size distributions requires knowledge about particle shape and density, CDMS serves as a highly complementary method to individual particle imaging‐based techniques. While image‐based analysis can be automated, the direct conversion of individual particle masses into diameters makes CDMS inherently well‐suited for high‐throughput sizing of large numbers of particles for improved characterization of particle size distributions. Sample preparation is also minimal, requiring only microliter volumes and simple solution dilution, offering a practical advantage over many other particle sizing methods. Although the diameters of the particles in this work are only a factor of two larger than prior accurate CDMS measurements of 100 nm particles, the masses are eight times higher. This represents a significant advance in the particle size where accurate masses can be measured. Large particles undergo aerodynamic acceleration in the standard atmospheric sampling interfaces of mass spectrometers that use metal capillaries to introduce ions into the mass spectrometer [67, 71]. This leads to particles that can have MeV and even GeV of kinetic energy, which makes them difficult to trap in CDMS [71]. Developments to reduce this high kinetic energy in order to extend these types of high‐accuracy mass measurements to even larger particles are ongoing.

A key strength of CDMS is the ability to resolve nanoparticle samples that differ only slightly in size. In this study, three samples spanning 191.4–205.9 nm in diameter were readily distinguished. The narrow distribution for the most homogenous sample indicates that size differences as small as 1%–1.5% could be resolved. Even finer discrimination could be achieved for samples with intrinsically narrower size distributions owing to the accuracy and resolution of the CDMS measurements. Together, these capabilities highlight CDMS as a powerful and scalable technique for precise nanoparticle characterization. Its high resolution, minimal sample requirements, and compatibility with automation make CDMS well‐suited for high‐throughput analysis of particles across many different fields where nanoscale particle properties are critical, including biomedicine, advanced electronics, and material science.

## Conflicts of Interest

The authors declare no conflicts of interest.

## Supporting information




**Supporting File**: smll74148‐sup‐0001‐SuppMat.pdf.

## Data Availability

The data that support the findings of this study are available in the supplementary material of this article.
