# Supplementary material for: Accurate Sizing and Resolution of Nominal 200 nm Diameter Polystyrene Nanospheres With Charge Detection Mass Spectrometry
Source: Small. 2026 Jun 12;22(43):e74148. doi: 10.1002/smll.74148 (PMC13432538; doi:10.1002/smll.74148)
Supplement: Supplementary file 1 — Supporting File: smll74148‐sup‐0001‐SuppMat.pdf. [file SMLL-22-e74148-s001.pdf]

Supplementary Information for

Accurate Sizing and Resolution of Nominal 200 nm Diameter Polystyrene Nanospheres with Charge Detection Mass Spectrometry

Veena S. Avadhani, Conner C. Harper, and Evan R. Williams*

Department of Chemistry, University of California, Berkeley, California, 94720-1460, United States

*Corresponding author

Email: erw@berkeley.edu

Table of Contents

Concentration of the Colloidal Metrics Sample………………………………………….... 3

Table S1………………………………………………………………………………….. 4

Hough Circle Transform………………………………………………………………… 5

Figure S1……………………………………………………………………………… 6

Table S2………………………………………………………………………………….. 7

Table S3………………………………………………………………………………….. 8

Figure S2………………………………………………………………………………… 9

Figure S3…………………………………………………………………………………10

Figure S4……………………………………………………………………………… 11

Figure S5……………………………………………………………………………… 12

Figure S6……………………………………………………………………………… 13

References……………………………………………………………………………….. 14

Concentration of the Colloidal Metrics Sample

The concentration of the Colloidal Metrics sample was not known. An estimate of this concentration was determined from the rate at which individual particles were detected from the three samples that were independently measured with CDMS. The particle detection rates for individual measurements of the three samples were approximately 16/min, 14/min, and 8/min for the Polyscience (P), Thermo Scientific (T), and Colloidal Metrics (CM) nanoparticles, respectively. Samples P and T were diluted so that their final concentrations used in the analysis were the same. The similar particle detection rates for the P and T samples indicate that these samples had similar ionization and transmission efficiencies. If this is true for the CM sample as well, the CM particle concentration was approximately half that of the other diluted samples corresponding to a stock solution concentration of ~3 x 10^{12} particles/mL (half that of P). This value is consistent with separate P + CM nanoparticle mixture experiment (Figure 4a), where the number of P particles is approximately two-fold higher than that of the CM particles.

**Specification**	**Parameters**
Inlet temperature	140 °C
Pressure in ion funnel chamber	3.5 Torr
Ion funnel front DC	320 V
Ion funnel back DC	280 V
Ion funnel exit lens	270 V
Ion funnel V_{pp}	280 V
Ion funnel RF	140 kHz
Gate valve entrance	250 V
Gate valve aperture	250 V
Rectilinear quad 1 DC offset	240 V
Rectilinear quad 1 asymptotic rods	300 V
Rectilinear quad 1 V_{pp}	600 V
Rectilinear quad 1 RF	45-50 kHz
Rectilinear quads 2+3 DC offset	230 V
Rectilinear quad 2 Asymptotic rods	245 V
Rectilinear quad 3 Asymptotic rods	235 V
Rectilinear quads 2+3 V_{pp}	850 V
Rectilinear quads 2+3 RF	36 kHz
Accelerator 1 upstream	0 V
Accelerator 1 downstream	120 V
Accelerator 2 upstream	0 V
Accelerator 2 downstream	25 V
Time between exit lens opening and trap entrance closing	2.9-3.1 ms
Trap time in quadrupole	100 ms
m/z filtering (quads)	~400,000

Table S1. Instrument parameters used for the CDMS measurements used to characterize 200 nm polystyrene beads. A detailed description of the instrument is provided elsewhere.[1]

Hough Circle Transform

To minimize manual measurement errors and efficiently analyze ~300+ nanoparticles, the Hough Circle Transform plugin in FIJI/ImageJ (version 1.53v) was employed.[2] The search radius was set between 85 and 176 pixels, and a Hough score threshold of 0.5 was used to identify valid circles. Each centroid overlap map generated by the transform was manually reviewed to ensure accurate identification and exclude any false detections. Figure S1 presents a representative TEM image of CM beads (a) and the corresponding overlay of the Hough transform on the original image (b). While the fitted circles may slightly underestimate the actual diameters, particularly for beads exhibiting darker edges extending beyond the fitted boundaries,[3] partial shrinking of the beads can cause minor deviations from sphericity, leading to such discrepancies.

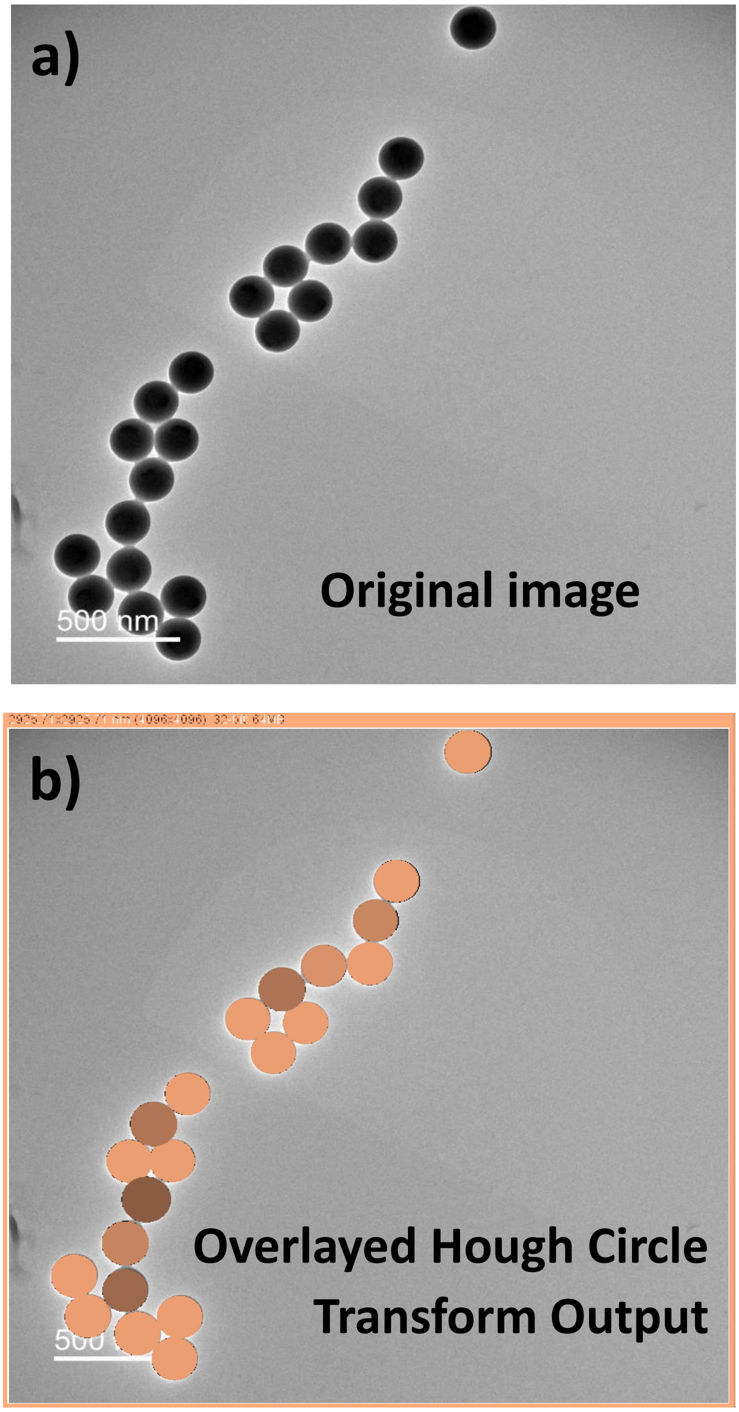

Figure S1. TEM image of CM polystyrene nanoparticles (a) along with overlaid colored circle fits determined using Hough Circle Transform function (b). The uneven dark edges extending out of the colored circles may be due to minor deviation from sphericity owing to PS bead shrinking under electron beam.

Technique	100 nm	200 nm
TEM	100 ± 7 (mean)[3]	200 ± 8 (Gaussian centroid) [*current study*]
CDMS	100 ± 7 (Gaussian centroid)[3]	199 ± 7 (Gaussian centroid) [*current study*]
DLS	112 ± 95 (Z-average)[4]	218 ± 103 (Z-average)[4]
NTA	105 ± 71 (mean)[4]	200 ± 71 (mean)[4]

Table S2. Measured sizes of 100 nm and 200 nm Thermo Scientific polystyrene nanoparticles obtained using different analytical techniques. Values represent means, Gaussian centroids, or Z-averages (intensity-weighted means) as specified. Reported ± values denote full-width-half-maxima (fwhm) of the distributions. For DLS data, fwhm values were estimated from polydispersity indices (PDI) provided by Filipe et al.[4] This was done using the relation: fwhm = mean diameter x 2.355 x $\sqrt{PDI}$. NTA distribution spreads were converted using fwhm = 2.355 x SD (standard deviation). These conversions were performed to enable a direct comparison of the distribution breadths across the different analytical platforms. TEM – Transmission Electron Microscopy; CDMS – Charge Detection Mass Spectrometry; DLS – Dynamic Light Scattering; NTA – Nanoparticle Tracking Analysis.

Diameters (nm)	P	T	CM
CDMS Mean (Fig d-f)	190.23	193.78	203.49
TEM 1 Mean (Fig g-i)	193.26	200.22	204.51
TEM 2 Mean (Fig j-l)	188.61	195.41	197.83
CDMS Gaussian centroid	191.42	199.13	204.75
TEM 1 Gaussian centroid	192.77	200.46	203.82
TEM 2 Gaussian centroid	187.14	195.51	197.09
CDMS Mode	191.7-191.8	200.2-200.3	206.4-206.5
TEM 1 Mode	193.37	200.486	204.752
TEM 2 Mode	189.59	196.72	196.72
CDMS Gaussian fwhm	2.95	7.01	1.83
TEM 1 Gaussian fwhm	4.61	8.06	4.67
TEM 2 Gaussian fwhm	5.47	6.39	6.82

Table S3. Mean, mode, centroid of Gaussian fit, and full-width-half-maximum (fwhm) of the Gaussian fit for CDMS and TEM measurements of P, T, and CM nanoparticles. TEM 1 and TEM 2 refer to TEM measurements corresponding to Figure 1 g-i and Figure 1 j-l, respectively.

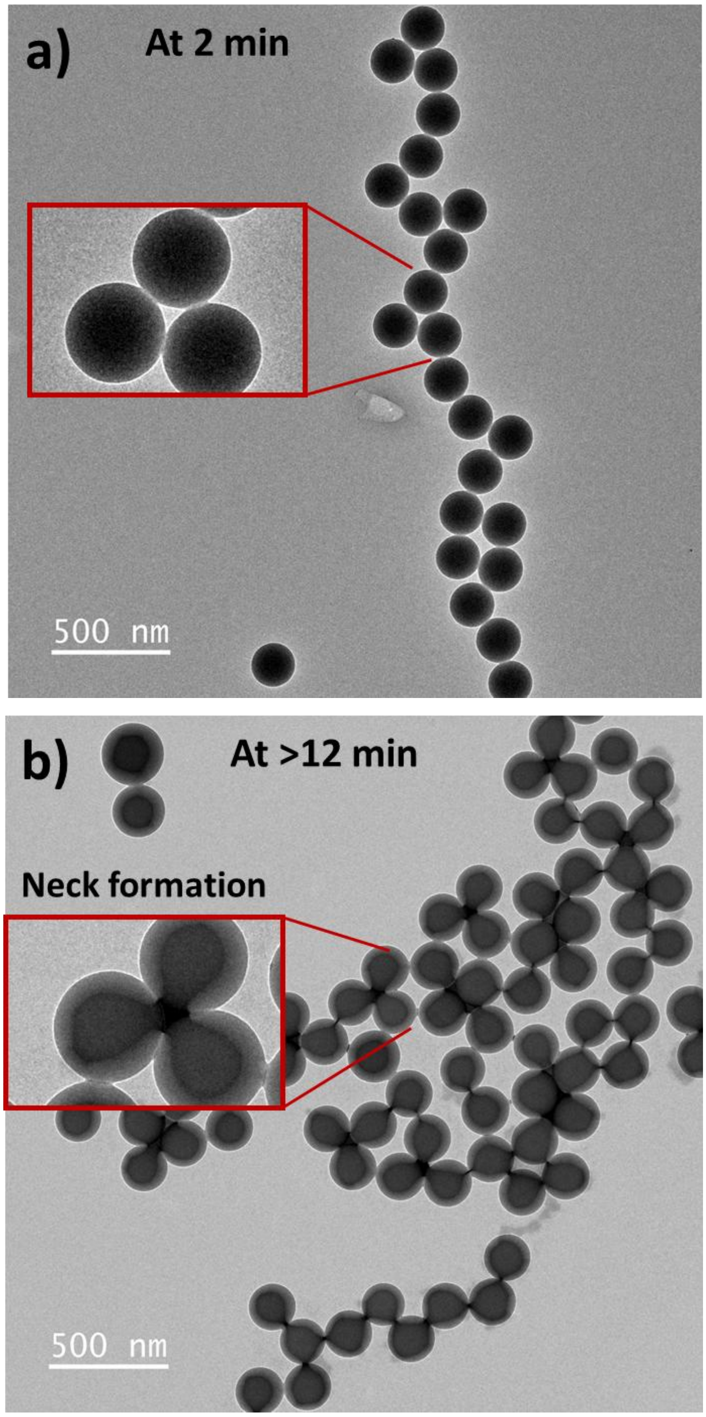

Figure S2. TEM images of CM nanoparticles captured (a) 2 min and (b) >12 min post electron beam irradiation. Zoomed inset in (b) shows neck formation between adjacent beads caused due to electron beam induced sintering as compared to a frame irradiated for lesser time, and thus, not impacted as in (a).

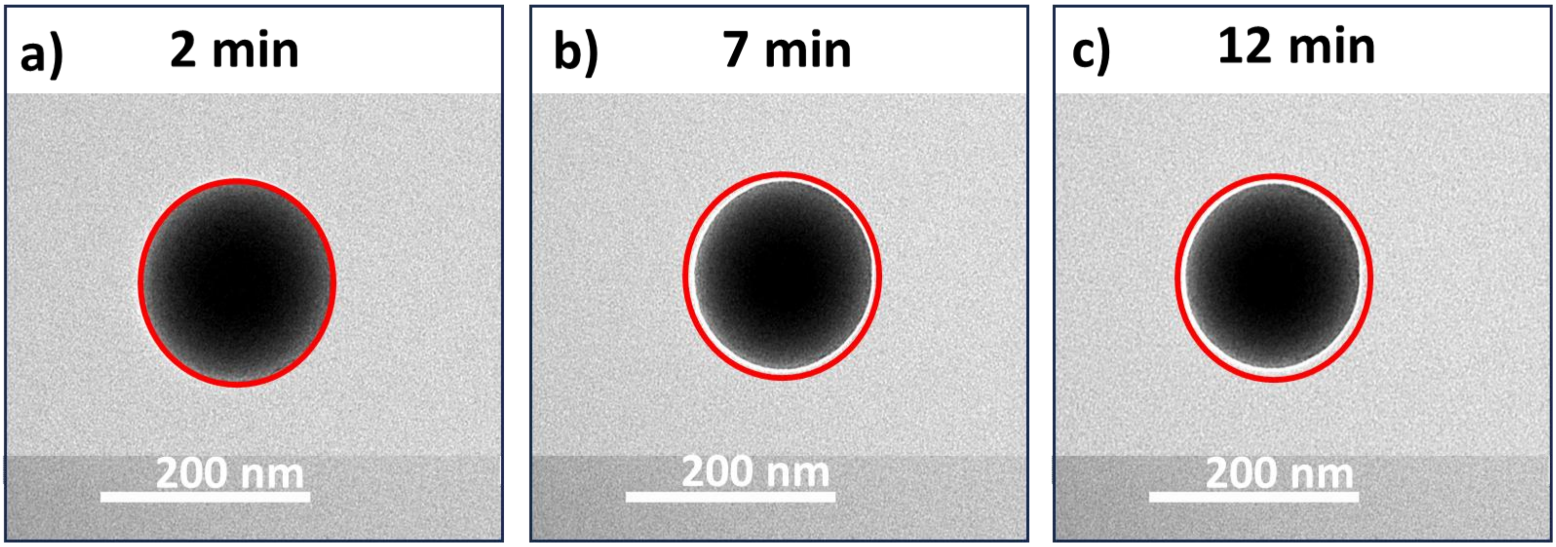

Figure S3. TEM images of the same P polystyrene bead in focus after electron beam irradiation for (a) 2 min, (b) 7 min, and (c) 12 min. The overlaid red circles of same size highlight shrinking of bead over the course of irradiation. Diameter of the bead shrunk from (a) 187.1 nm to (b) 174.3 nm to (c) 166.3 nm. These results are analogous to results shown in Figure 2, albeit milder beam condition with spot size 5 (instead of 2) is being used. Increase in spot size translates to lesser number of electrons incident per unit area.

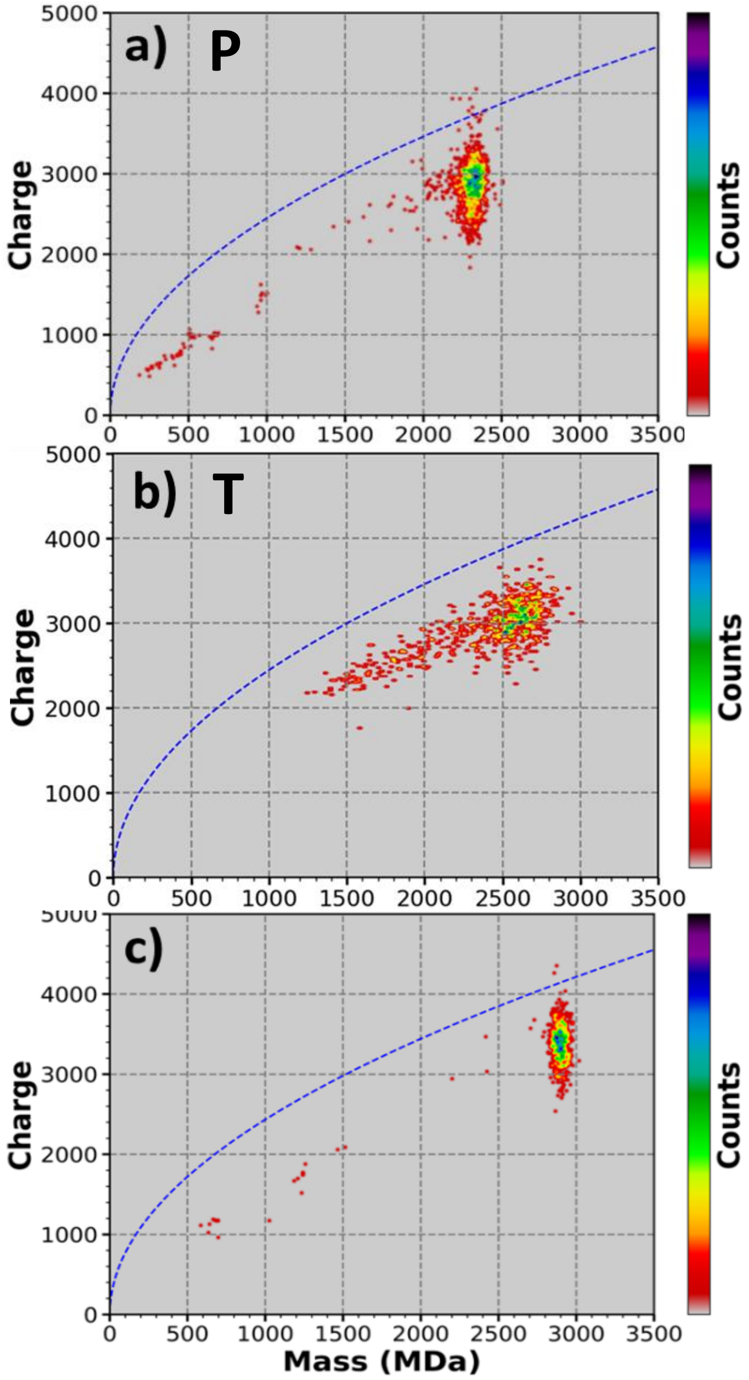

Figure S4. Two-dimensional mass vs charge plots for (a) P, (b) T, and (c) CM polystyrene nanoparticles. The blue line represents Rayleigh charge limit for a water droplet of given mass. P beads have a charge centroid at 2888 *e*, T at 2999 *e*, and CM at 3392 *e*. All the three types of nanoparticles are charged at ~77% of the Rayleigh limit.

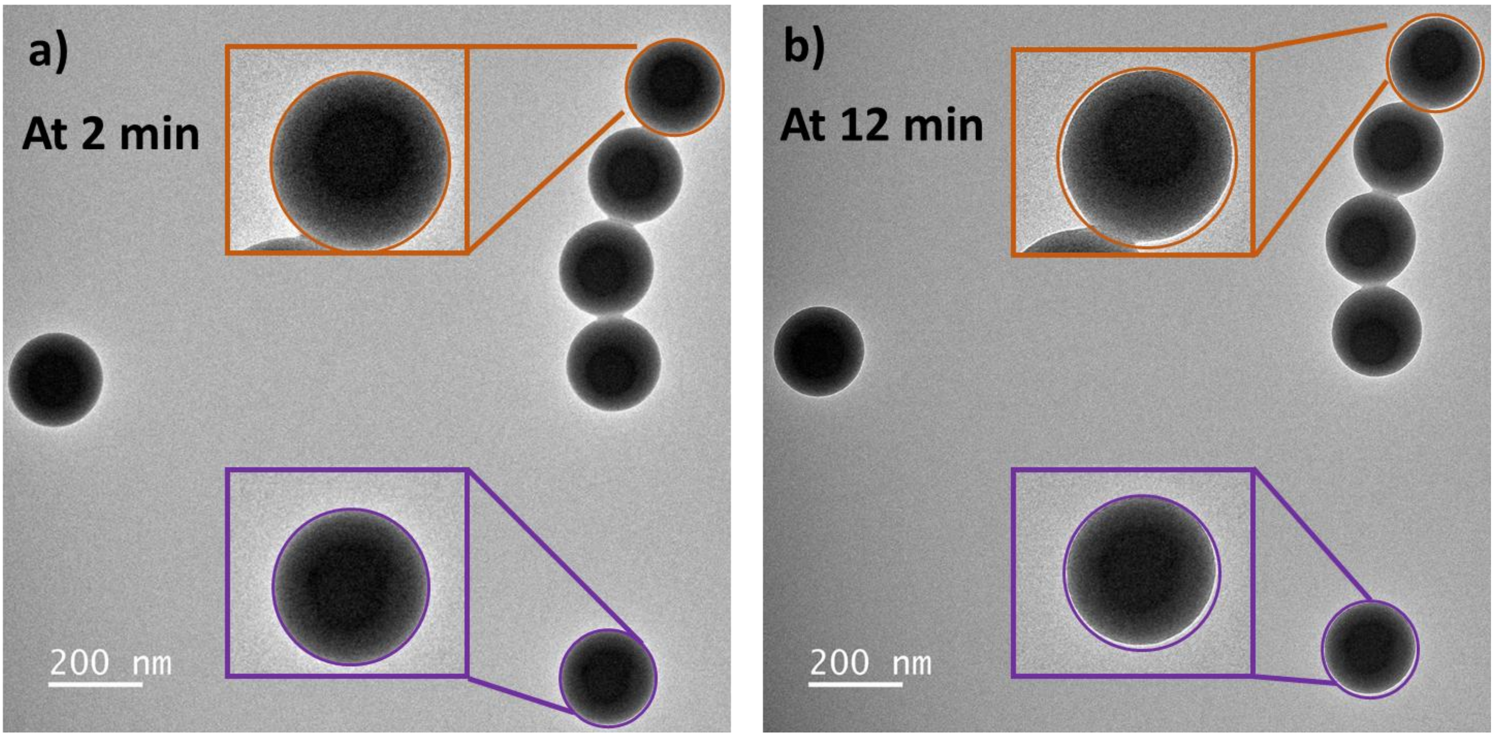

Figure S5. TEM images of T polystyrene beads captured at (a) 2 min and (b) 12 min after electron beam irradiation. The orange encircled bead shrunk by 3.5% in diameter while the purple encircled bead shrunk by 5.2%. This example highlights the varying degree of shrinking that happens for individual particles and affect the TEM-based size measurements.

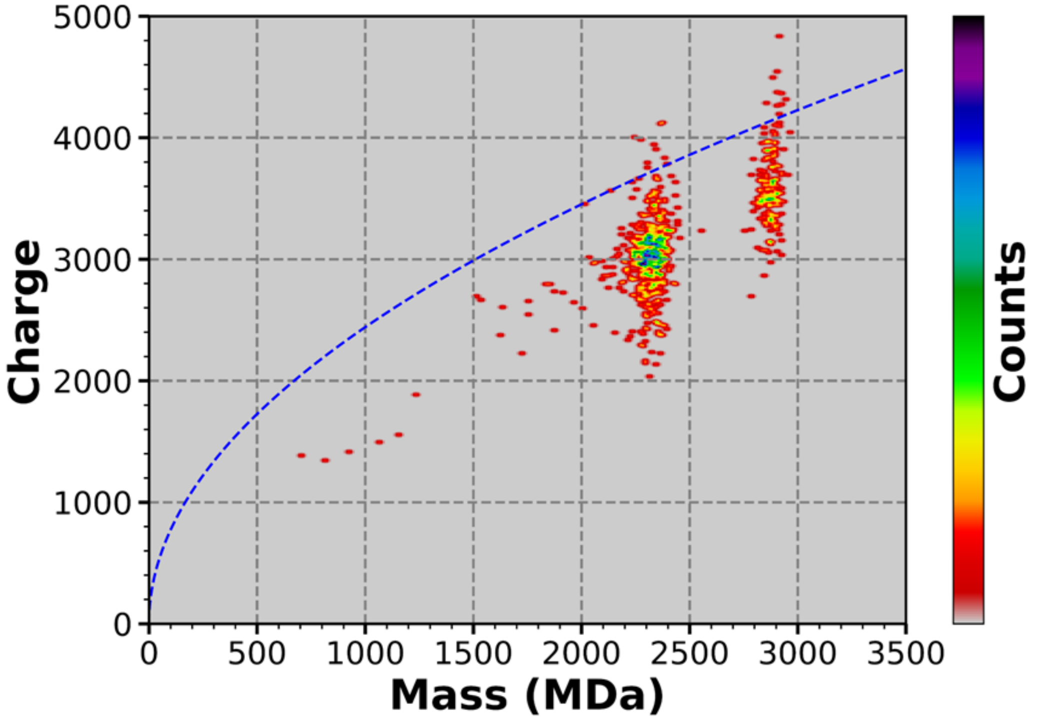

Figure S6. Two-dimensional mass vs charge histogram of a mix of P and CM polystyrene nanoparticles representing baseline resolution achieved by CDMS. The blue line represents Rayleigh charge limit for a water droplet of given mass. P beads have a charge maximum at 2888 *e* and CM at 3392 *e*. Both types of nanoparticles are charged at ~77% of the Rayleigh limit.

References

(1) Harper, C. C.; Miller, Z. M.; McPartlan, M. S.; Jordan, J. S.; Pedder, R. E.; Williams, E. R. Accurate Sizing of Nanoparticles Using a High-Throughput Charge Detection Mass Spectrometer without Energy Selection. *ACS Nano* **2023**, *17* (8), 7765–7774. https://doi.org/10.1021/acsnano.3c00539.

(2) *Hough Circle Transform*. MediaWiki. https://imagej.net/plugins/hough-circle-transform (accessed 2025-10-21).

(3) Harper, C. C.; Jordan, J. S.; Papanu, S.; Williams, E. R. Characterization of Mass, Diameter, Density, and Surface Properties of Colloidal Nanoparticles Enabled by Charge Detection Mass Spectrometry. *ACS Nano* **2024**, *18* (27), 17806–17814. https://doi.org/10.1021/acsnano.4c03503.

(4) Filipe, V.; Hawe, A.; Jiskoot, W. Critical Evaluation of Nanoparticle Tracking Analysis (NTA) by NanoSight for the Measurement of Nanoparticles and Protein Aggregates. *Pharm. Res.* **2010**, *27* (5), 796–810. https://doi.org/10.1007/s11095-010-0073-2.
